# Therapeutic and diagnostic challenges for frontotemporal dementia

**DOI:** 10.3389/fnagi.2014.00204

**Published:** 2014-08-19

**Authors:** Simon D’Alton, Jada Lewis

**Affiliations:** Department of Neuroscience, Center for Translational Research in Neurodegenerative Disease, College of Medicine, University of FloridaGainesville, FL, USA

**Keywords:** frontotemporal dementia, frontotemporal lobar degeneration, TDP-43, tau, therapeutics

## Abstract

In the search for therapeutic modifiers, frontotemporal dementia (FTD) has traditionally been overshadowed by other conditions such as Alzheimer’s disease (AD). A clinically and pathologically diverse condition, FTD has been galvanized by a number of recent discoveries such as novel genetic variants in familial and sporadic forms of disease and the identification of TAR DNA binding protein of 43 kDa (TDP-43) as the defining constituent of inclusions in more than half of cases. In combination with an ever-expanding knowledge of the function and dysfunction of tau—a protein which is pathologically aggregated in the majority of the remaining cases—there exists a greater understanding of FTD than ever before. These advances may indicate potential approaches for the development of hypothetical therapeutics, but FTD remains highly complex and the roles of tau and TDP-43 in neurodegeneration are still wholly unclear. Here the challenges facing potential therapeutic strategies are discussed, which include sufficiently accurate disease diagnosis and sophisticated technology to deliver effective therapies.

## Introduction

Frontotemporal dementia (FTD) is the third most common dementia in modern society, rendering it a critical public health issue (Onyike and Diehl-Schmid, 2013). However there is currently a dearth of therapeutics in clinical trials that are intended for FTD. Although tau has long been recognized as the principle component of neurofibrillary tangles (NFTs) in FTD (Joachim et al., 1987) and mutations in the microtubule associated protein tau (*MAPT*) are responsible for a subset of FTD cases (Hutton et al., 1998; Spillantini et al., 1998; D’Souza et al., 1999), investment in tau therapies has traditionally lagged somewhat due to the focus on proteotoxicity of other aggregated proteins such as amyloid-beta (Aβ) in Alzheimer’s disease (AD; Schneider et al., 2014). Furthermore, the majority of advances in genetics—so often a guiding hand for neurodegenerative research in the identification of therapeutic targets—have occurred only relatively recently in FTD (Hutton et al., 1998; Watts et al., 2004; Baker et al., 2006; Cruts et al., 2006; Sreedharan et al., 2008; DeJesus-Hernandez et al., 2011; Renton et al., 2011), and have uncovered genes such as progranulin (*GRN*) and Tar DNA binding protein (*TARDBP*) with little preexisting neurological literature. For example, mutation of the gene *TARDBP* is pathogenic in amyotrophic lateral sclerosis (ALS; Sreedharan et al., 2008) and the encoded protein TAR DNA binding protein of 43 kDa (TDP-43) has been identified as a major component of the ubiquitinated inclusions that characterize approximately half of all FTD cases (Taniguchi et al., 2004; Shi et al., 2005; Neumann et al., 2006); however, only 12 publications on this protein existed prior to this discovery.

Findings such as these can drive promising therapeutic research. Our understanding of the clinical symptoms, neuropathological features and genetic contributions to FTD has vastly expanded. At this point, we should be asking if we have reached the critical mass of knowledge necessary to develop effective therapeutics for clinical trials and, if not, we must assess what the remaining hurdles are for therapeutic efforts to have the highest probability of success. The ability to do so will depend not only on developing bioactive compounds, but also on sufficiently accurate diagnosis to determine those persons in early, if not asymptomatic, stages of degenerative disease. While strides are being made in many of these areas, there are still numerous challenges that must be met prior to effective therapeutic intervention to treat this chronic, multifaceted condition. Fortunately, lessons learned from the failures of clinical trials in neurodegenerative diseases such as AD can inform clinical efforts for FTD.

## Current pharmaceutical intervention in FTD

The current trialed therapy to date for FTD is based on management of symptoms and does not address cause, and the rationale for their use is based on efficacy in treating other neurodegenerative disorders or psychiatric conditions with similar behavioral phenotypes. All of the clinical trials have been small, and only a handful has been placebo-controlled, double blind trials (Rabinovici and Miller, 2010). Generally, there is some evidence to suggest that selective serotonin reuptake inhibitors or serotonin norepinephrine reuptake inhibitors may be beneficial for some patients, particularly those with behavioral disturbances (Boxer and Boeve, 2007; Vossel and Miller, 2008). Antipsychotics and drugs targeting the cholinergic system appear to be largely ineffective (Mendez et al., 2007). Furthermore, despite its off-label use to treat FTD, the largest clinical study to date found no benefit to patients of taking memantine (Boxer et al., 2013). Consequently, there is no definitive treatment for FTD, and there is a dire need for novel therapeutics that target the underlying causes.

## Clinical FTD and pathological FTLD

### Clinical and pathological heterogeneity of FTD/FTLD

FTD is a clinical term for a cluster of syndromes resulting from degeneration of the frontal and temporal lobes. Although historically regarded as a disorder with presenile onset (prior to 65 years of age), perhaps 25% of pathologically confirmed cases present clinically later than this (Baborie et al., 2011; Onyike and Diehl-Schmid, 2013).

Several syndromes can present in FTD which are associated with specific regional atrophy (Kril et al., 2005; Pereira et al., 2009; Seelaar et al., 2011). Behavioral-variant frontotemporal dementia (bvFTD) is characterized by changes in personality and behavior, and patients are apathetic (loss of interest in responsibilities, social withdrawal) and disinhibited (demonstrating socially inappropriate behavior) (Neary et al., 1998; Rascovsky et al., 2011). Semantic dementia (SD) and progressive nonfluent aphasia (PNFA) comprise the two syndromes that are characterized by changes in language function. SD presents with impaired comprehension and anomia, in contrast to PNFA in which language comprehension is spared, but speech is effortful and grammatically erroneous (Gorno-Tempini et al., 2011). These three variants often overlap, particularly at later stages of disease as atrophy becomes more widespread (Mesulam et al., 2003; Marczinski et al., 2004; Banks and Weintraub, 2008). The FTD umbrella also includes corticobasal syndrome (CBS) and progressive supranuclear palsy syndrome (PSPS; Boeve et al., 2003; Josephs, 2008), which while originally identified as atypical movement disorders also frequently present with features of bvFTD or PNFA. Similarly, individuals initially showing typical bvFTD/PNFA can develop motor disturbances (Rabinovici and Miller, 2010). bvFTD is frequently accompanied by an element of motor dysfunction as in motor neuron disease (FTD-MND) (Geser et al., 2010). Thus, the clinical picture of FTD is a spectrum of overlapping syndromes.

Underlying these clinical syndromes is a cluster of diseases collectively termed frontotemporal lobar degeneration (FTLD). In keeping with the historical approach to neurodegenerative conditions, definitive diagnosis of underlying disease is reserved for postmortem analysis based on pathological findings (i.e., the nature of the protein aggregate in specific brain regions). Pathologically, FTLD can now be broadly split into two major categories; FTLD-TDP with neuronal and glial inclusions immunoreactive for TDP-43, and FTLD-tau containing fibrillar, hyperphosphorylated tau (Joachim et al., 1987; Cairns et al., 2007; Neumann et al., 2007a). A small number of tau-negative, TDP-43-negative cases comprise the remainder which are immunoreactive to components of the ubiquitin proteasome system (FTLD-UPS), the fused in sarcoma protein (FTLD-FUS), ubiquitin only (FTLD-U) or lack inclusions completely (FTLD-ni/FTLD-other) and which will not be discussed here (Holm et al., 2009; Urwin et al., 2010; Josephs et al., 2011).

### Heterogeneity of FTLD-tau

Further complexity of FTLD is evident in the nature of the TDP-43 or tau inclusions themselves, which appear as a variety of subtypes. In the case of tau lesions, FTLD-tau can be subdivided further to CBD (Dickson et al., 2002), progressive supranuclear palsy (PSP; Hauw et al., 1994), Pick’s disease (PiD; Dickson, 1998), argyrophilic grains disease (AGD; Braak and Braak, 1987, 1989), sporadic multisystem tauopathy (MST; Bigio et al., 2001) and diffuse NFT dementia with calcifications (DNTC; Kosaka, 1994). PSP, CBD and PiD comprise by far the majority of cases of FTLD-tau (Josephs et al., 2011). In addition to these sporadic diseases, FTD and Parkinsonism linked to chromosome 17 (FTDP-17*t*) is caused by mutations in *MAPT*—this genetic form representing the cornerstone piece of evidence for the involvement of tau in non-familial disease (Hutton et al., 1998; Spillantini et al., 1998; D’Souza et al., 1999). Each of these conditions is characterized by the appearance of hyperphosphorylated tau that self-aggregates into fibrillar paired helical filaments (PHFs) and consequently neurofibrillary tangles (NFTs), although the morphology of the inclusion differs amongst diseases. Some of this heterogeneity is derived from alternative splicing of exon 10 of *MAPT*, which encodes one of the four “repeat” regions that act as microtubule binding domains (Goedert et al., 1989; Kosik et al., 1989). The deposited tau in PiD is rounded in nature and cytoplasmic, and is unique in that it primarily contains only 3 of the repeats (exclusion of exon 10, “3R” tau) (Delacourte et al., 1998; de Silva et al., 2006). Inclusions of PSP and CBD are both primarily composed of tau containing exon 10 and thus all four repeat regions (“4R” tau) (Sergeant et al., 1999). Using numerous antibodies to phosphorylated epitopes of tau, it has been proposed that the antigenicity of inclusions in PSP and CBD are similar to one another yet distinct from other tauopathies such as AD (Berry et al., 2004). Subtle differences between the two may be discerned on the basis of the nature of the inclusion. Neuronal inclusions in PSP are large and globose, and are accompanied by astrocytic lesions termed “tufted astrocytes”; whereas, CBD is characterized by more rounded “cortico-basal bodies” in neurons and “astrocytic plaques” (Yamada et al., 1992, 1993; Dickson et al., 1996; Josephs et al., 2011). It is worth reiterating here that there is a distinction between the pathological terms such as FTLD, CBD and PSP and the clinical terms FTD, CBS and PSPS as they are often incorrectly used interchangeably and can lead to confusion if, for example, CBD or PSP is the underlying pathological disease in a clinical case of PNFA (Josephs et al., 2011 and see also “Current Clinical, Imaging and Biochemical Diagnostics” section below on correlation between clinical symptom and neuropathologically diagnosed disease).

### Heterogeneity of FTLD-TDP

A major component of inclusions in cases of FTLD with ubiquitinated aggregates, which represents approximately 50% of FTLD cases, was identified in 2006 to be TDP-43, giving rise to the name FTLD-TDP (Neumann et al., 2006). There are four subtypes of FTLD-TDP, associated with distinct TDP-43 pathological morphology and distribution—Type A is characterized by many aggregated, juxtanuclear, neuronal cytoplasmic inclusions (NCI) and short dystrophic neurites (DN) predominantly in layer 2 of the cortex, Type B by fewer NCI and DN that are dispersed more widely throughout the cortex, Type C by long DN in cortical layer 2 and Type D by the appearance of neuronal intranuclear inclusions (Mackenzie et al., 2011). Type D is typically found in rare, familial cases involving mutations in the valosin containing protein (*VCP*) gene (Forman et al., 2006), thus the defining hallmark of sporadic FTLD-TDP is cytoplasmic aggregation as NCI or DN. This is accompanied by a loss of nuclear TDP-43 expression (Neumann et al., 2006). In FTLD-TDP, TDP-43 is also hyperphosphorylated and truncated to produce ~25 kDa C-terminal fragments, which appear to be enriched in inclusions relative to the N-terminus (Igaz et al., 2008). Therefore, as is the case for FTLD-tau, TDP-43 neuropathology appears as a number of distinct entities and pathological subtypes. The toxicity of TDP-43 and tau in disease is not evident from neuropathology alone, and critical evidence for the involvement of both in disease hinge on compelling genetic data.

## FTLD genetics as a driver of translational research

Studies of the genetic component of FTLD have provided important breakthroughs in our understanding of disease pathogenesis. As noted above, familial mutations in the *MAPT* gene encoding the tau protein explain a subset of FTLD cases and provide compelling evidence for the involvement of tau in sporadic disease, which share some of the same pathological hallmarks (Hutton et al., 1998; Spillantini et al., 1998; D’Souza et al., 1999). Whilst inherited mutations in the *TARDBP* gene encoding TDP-43 are not found in heritable FTLD, familial cohorts do exist in ALS, a second TDP-43 proteinopathy (Sreedharan et al., 2008), implicating TDP-43 in neuronal dysfunction or death. This is used, in a manner analogous to that for tau and AD, as evidence that TDP-43 dysfunction is a driver of FTLD also. Since ALS and FTD are now considered at opposite ends of the same disease spectrum, the connection seems justified on a biological level also. Adding further weight to this argument are reports of *TARDBP* mutations in sporadic FTLD (Borroni et al., 2009a, 2010; Synofzik et al., 2014).

Interestingly, familial cohorts do exist for TDP-43 positive FTLD, although the mutations in this case are in the *VCP*, *C9ORF72* and *GRN* genes (Watts et al., 2004; Baker et al., 2006; Cruts et al., 2006; Neumann et al., 2007b; DeJesus-Hernandez et al., 2011; Renton et al., 2011). From a therapeutic standpoint, by far the most intriguing of these is the secreted glycoprotein progranulin, primarily due to its neurotrophic activity (Van Damme et al., 2008). It also has roles in the periphery including in inflammation and wound healing (He et al., 2003; Ahmed et al., 2007). *GRN* mutations can be insertions, deletions, splice site, missense or nonsense and are scattered throughout the gene (Baker et al., 2006; Cruts et al., 2006; Gass et al., 2006; Mukherjee et al., 2006; Bronner et al., 2007; Bruni et al., 2007; Rademakers and Rovelet-Lecrux, 2009). However, all are predicted to result in reduction of functional progranulin (Baker et al., 2006; Cruts et al., 2006; Rademakers and Rovelet-Lecrux, 2009). Loss of progranulin function in these familial cases causes type A TDP-43 pathology (Mackenzie et al., 2006). Common genetic variants in progranulin are also potential risk factors for TDP-43 proteinopathies including sporadic FTLD (Brouwers et al., 2008; Sleegers et al., 2008; Galimberti et al., 2010). Currently, mechanisms by which reduction in progranulin results in neurodegeneration are unclear but principally center on a reduced ability to recover from age-related stressors or damage (Ryan et al., 2009; Laird et al., 2010; Kao et al., 2011; Jackman et al., 2013).

The discovery of mutations in *GRN* in 2006 left only one linkage region on chromosome 9 unaccounted for in hereditary cases of FTD. The delay in identifying the responsible *C9ORF72* gene can be attributed to the nature of the mutation itself—an intronic, hexanucleotide (GGGGCC) repeat expansion that was undetectable by commonly used sequencing methods (DeJesus-Hernandez et al., 2011; Renton et al., 2011). Both the antisense and sense strands are transcribed from this genomic locus and form nuclear RNA aggregates or “RNA foci” (DeJesus-Hernandez et al., 2011; Gendron et al., 2013; Zu et al., 2013), which may cause cell death via sequestration of functional RNA binding proteins (Lee et al., 2013b). The expanded RNA also undergoes repeat-associated non-ATG translation (RAN translation) to produce aggregation prone polypeptides that form inclusions in human FTD (Ash et al., 2013; Gendron et al., 2013; Mori et al., 2013; Zu et al., 2013). Reducing levels of expanded transcripts is an obvious therapeutic concept and one that has already proven successful *in vitro* (Donnelly et al., 2013). Another group has demonstrated antisense efficacy in motor neurons derived from *C9ORF72* carriers (Sareen et al., 2013). However, future studies that clarify the mechanistic link between RNA foci, RAN proteins, and neurodegeneration will be essential in order to determine if such approaches are likely to fully address the neurotoxicity in *C9ORF72* cases. It should be also noted that the neuropathology unique to *C9ORF72* is not present in FTD lacking repeat expansion mutations. Although *C9ORF72* mutations constitute a fascinating additional mechanism by which genetic aberration induces neuronal demise, therapeutic implications for FTD cannot currently be extrapolated to the population at large.

In addition to these familial, causative mutations, there are a number of genes that modulate the risk of FTD or phenotypic outcome. Notably, homozygous mutations in *TREM2* are associated with atypical FTD presenting with epilepsy and parkinsonism (Giraldo et al., 2013; Guerreiro et al., 2013; Le Ber et al., 2014). Rare heterozygous mutations in this gene may increase risk of FTD (Lattante et al., 2013; Borroni et al., 2014; Cuyvers et al., 2014). Several genes identified in genome wide association studies may also modify risk (Ferrari et al., 2014).

## Tau and tau-mediated mechanisms of toxicity

### Tau biology and function

The *MAPT* gene generates six isoforms of the microtubule binding protein tau via alternative splicing of exons 2, 3 and 10 (Kosik et al., 1989). Exon 10 contains one of four microtubule binding domains in tau, such that exon inclusion generates the 4R isoform with higher affinity than 3R for microtubule binding, these two forms being expressed at approximately 1:1 ratio in human adult brain (Goedert et al., 1989; Goedert and Jakes, 1990; Butner and Kirschner, 1991). The binding of tau stabilizes microtubules and promotes assembly (Weingarten et al., 1975; Cleveland et al., 1977). Under physiological conditions in healthy neurons, this activity is further regulated by phosphorylation of tau at 47 confirmed sites (Martin et al., 2013) by a variety of major cellular kinases including glycogen synthase kinase 3 beta (GSK-3β; Wagner et al., 1996), microtubule affinity regulating kinase (MARK; Drewes et al., 1997) cyclin dependent kinase 5 (CDK5; Baumann et al., 1993), Protein Kinase A and C (PKA/PKC; Michel et al., 1998) and by phosphatases such as protein phosphatase 2a (PP2A; Sontag et al., 1996). Furthermore, our lab has recently demonstrated that the Parkinson’s disease associated kinase LRRK2 can phosphorylate tau (Bailey et al., 2013). Tau is regulated both in normal and disease states by phosphorylation, with phosphorylated tau having reduced affinity for microtubules, preventing their stabilization (Drechsel et al., 1992).

Although microtubule binding is the major function ascribed to tau, it has a number of other roles, for example in influencing cell signaling via kinases such as Fyn, Src (Sharma et al., 2007) and phospholipase Cγ (Hwang et al., 1996). A nuclear form of tau is also implicated in protection from stress (Sultan et al., 2011), indicating that our current knowledge of tau function is probably incomplete. Additionally, our knowledge of “what” tau does is often interpreted without the context of “when”, an important consideration when aiming to modify any activity but particularly so in an age-related condition. For example, there are numerous external inputs that may induce tau phosphorylation, including oxidative stress (Melov et al., 2007), hypoxia (Fang et al., 2010), hypothermia (Planel et al., 2007a), administration of drugs (Whittington et al., 2011), insulin dysfunction (Planel et al., 2007b) and viral infection (Patrick et al., 2011). Splice exclusion of tau exon 10 also occurs in response to hypoxia (Suh et al., 2010). These observations imply that tau is dynamically regulated in response to external cues, which may be important when evaluating scientific theories and therapeutic avenues.

### Tau aggregation and toxicity

**Figure 1:**
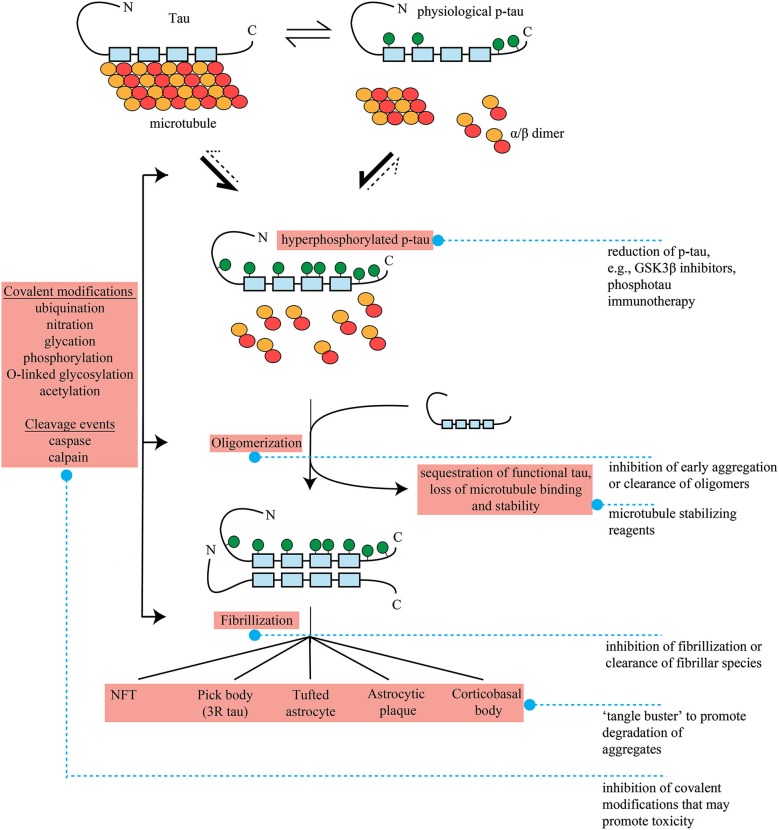
**Formation of pathological tau and potential therapeutic interventions in FTLD**. Tau bound to microtubules through repeat regions (blue boxes, 4 repeat tau shown) exists in dynamic equilibrium with physiological, phosphorylated tau (p-tau). P-tau formation is accompanied by microtubule disengagement and depolymerization to tubulin heterodimers. The point of equilibrium between tau and p-tau is influenced by genetics and external factors such as oxidative stress and hypothermia as well as stages of development. It is unclear if hyperphosphorylated, disease-associated tau results from tipping of this equilibrium to favor further p-tau formation, completely novel phosphorylation pathways, or a mixture of the two. In the hyperphosphorylated state, microtubule destabilzation beyond physiological levels results in neuronal dysfunction. This loss of function may be enhanced further by sequestration of functional tau by hyperphosphorylated tau into oligomers, and subsequently fibrils and aggregates. The repeat region of tau appears to form the core of these insoluble species. A number of other covalent modifications (ubiquination, nitration, glycation, phosphorylation, O-linked glycosylation) and cleavage events (caspase and calpain) may also occur at ill-defined junctures, and these may potentiate the dynamics of oligomerization or aggregate formation. As there are a range of pathological tau lesions which differ in appearance, these and other undefined pathways and potentially factors such as genetics, environmental inputs and cell type specific expression must differ in pathogenic tau formation. Pick bodies are entirely three repeat tau. Pathogenic species or potentially toxic mechanisms related to tau are highlighted in red. Blue dashed lines denote potential therapeutic interventions, see main text for more detail.

The identification of which form(s) of tau are actually toxic remains the largest question in the field, and is a key determinant of future therapeutic avenues. Originally, tangles themselves or smaller fibrillar antecedents were considered the species most likely to be neurotoxic, and theories regarding direct toxicity via physical blockade of cellular function such as trafficking, disruption to organelle distribution and inhibition of proteasome activity remain intuitive and often well-researched hypotheses (Figure 1; Keck et al., 2003; Lin et al., 2003; Ren et al., 2007).

Despite this, several lines of evidence weigh against tangles as a form of tau toxicity, or at least suggest that they are not solely responsible. For example, neurons survive for perhaps decades with neurofibrillary pathology (Morsch et al., 1999) and silencing of the tau transgene in a mouse model of tauopathy following formation of NFT pathology rescues cognitive phenotype, although tangle formation continues unabated (Santacruz et al., 2005). Furthermore, several transgenic lines of mice and tau transgenic flies expressing various tau isoforms display a distinct dissociation between cell death and the appearance of tangle pathology (Wittmann et al., 2001; Andorfer et al., 2005; Spires et al., 2006). In fact, tangle-bearing neurons in mice expressing mutant P301L tau appear to be functionally identical to both wild type mice and to neighboring neurons that do not contain NFTs (Kuchibhotla et al., 2014). Additionally, study of human disease has demonstrated that despite greater tissue loss in FTDP-17*t*, the aggregated tau burden in these cases is approximately 10% of the level of that found in AD (Shiarli et al., 2006). While these studies do not exclude the possibility that tangles are ultimately toxic, they do suggest that tangles are not the initial neurotoxic species. Rather, such findings have shifted philosophy towards a less visible form of pre-fibrillar species, with aggregated material representing compensatory adaptation to sequester these smaller toxic forms, at least initially. Several groups have reported the existence of what might be referred to as tau oligomers (Berger et al., 2007; Maeda et al., 2007; Sahara et al., 2007). Although the nature and importance of these species are contentious, several studies have demonstrated an association between their levels and neurotoxicity. For example, heat-shock protein mediated stabilization of oligomers in the rTg4510 model of tauopathy is associated with enhanced neurotoxicity but reduced fibril formation (Blair et al., 2013). Conversely, immunotherapy in the JNPL3 transgenic mouse line of tauopathy using an antibody to oligomeric forms of tau improves motor and memory phenotypes without altering NFTs (Castillo-Carranza et al., 2014).

### Post-translational modifications, hyperphosphorylation and tau loss-of-function

Tau modifications that occur prior to or concomitant with fibril formation or oligomerization may potentiate toxicity. These processing events include cleavage by caspases (Canu et al., 1998; Rohn et al., 2002) and calpain (Park and Ferreira, 2005; Ferreira and Bigio, 2011), O-linked glycosylation (Arnold et al., 1996; Liu et al., 2004), ubiquitination (Morishima-Kawashima et al., 1993), nitration (Horiguchi et al., 2003; Reyes et al., 2012), acetylation (Min et al., 2010) and glycation (Ledesma et al., 1994; Yan et al., 1994); however, the timing and significance of these modifications in pathogenesis is frequently debated (reviewed in Gendron and Petrucelli, 2009). The addition of such moieties and/or proteolytic cleavage have been generally linked in some way to either facilitation of tau into fibrillar species, the enhancement of subsequent events such as phosphorylation or to modification of function which might therefore facilitate the evolution of tau from functional molecule to pathological toxin.

Of these, tau hyperphosphorylation is by far the most cited candidate for a mediator of toxicity. Although phosphorylation has been implicated in facilitation of fibril formation and aggregation, which appears to differ between phosphorylation sites (Alonso et al., 1996; Necula and Kuret, 2004, 2005; Wang et al., 2007), pseudophosphorylated tau is also toxic in cell culture without the need for fibril formation, demonstrating a pathogenic effect of phospho-tau without fibrillization (Fath et al., 2002). This has driven an alternative hypothesis that abnormal phosphorylation of tau results in inappropriate disengagement from microtubules and consequent destabilization, which in turn results in defects such as reduced axonal transport—in essence, a tau loss of function. The absence of any robust neurodegenerative phenotype in tau knockout mice may contradict this hypothesis, although it seems plausible that constitutive loss of tau may be compensated for by *in utero*-programmed upregulation of other microtubule binding proteins such as MAP1a (Harada et al., 1994). Interestingly, the absence of phenotype in this mouse is also often cited as evidence that a potential therapy which reduces total tau, and therefore any toxic species, would not yield adverse consequences. The simultaneous assertion that loss of function is a possible mechanism of disease and that reduction in total tau should be considered for tau therapeutics is both highly contradictory and highlights the current lack of clarity regarding disease mechanism(s). In this specific instance, a conditional tau knockout mouse may provide greater insight.

Nevertheless, loss of microtubule stability remains a plausible neurodegenerative mechanism and phosphorylation of tau is a proven destabilizer. Hyperphosphorylated tau extracted from AD, another tauopathy, is a poor promoter of microtubule assembly and sequesters normal functional tau and other microtubule binding proteins *in vitro* (Alonso et al., 1996, 1997). Importantly, this effect is lost upon fibrillization or treatment with phosphatases (Wang et al., 1996; Alonso Adel et al., 2006). Activation of cdk5 *in vivo* using overexpression of p25 induces tau phosphorylation with accompanying features characteristic of loss of microtubule integrity (Ahlijanian et al., 2000; Bian et al., 2002). Genetic evidence also supports the tau loss of function and microtubule destabilization hypothesis. Mutations in *MAPT* that are the cause of FTDP-17*t* typically are either intronic and alter the splicing of exon 10 encoding one of the microtubule binding domains or cause changes in coding sequences situated close to the microtubule binding domain (Goedert, 2005). This suggests that correct splicing of tau and consequently tau-mediated microtubule stability is an important part of healthy aging. In many of these familial cases, the aggregated tau is almost entirely of the 4R form (Spillantini et al., 1997).

From the evidence above, it can be concluded that tau undergoes a host of conformational and biochemical changes indicating that in all likelihood there are a wide range of pathways acting upon it at a number of different junctures (Figure 1). During this evolution, tau may be toxic at one or several points via a number of different mechanisms that may occur simultaneously, and there is currently evidence supporting and undermining each.

## Hypothetical therapeutic strategies for FTLD-tau

The critical issues surrounding toxicity pose significant confounds for the development of therapeutic approaches for FTLD-tau. Hypothetical therapeutic strategies are at present based on a highly complex toxicity that is not fully understood. At this time, none of the aforementioned toxic mechanisms has emerged as the dominant candidate in the push for therapy development. Consequently, although there are a number of potential therapeutic possibilities (Figure 1) there is no clear cut candidate above all others for therapeutic intervention.

Under the gain of function aggregate toxicity model, determining toxic species will be highly influential in future direction. For example, the removal of NFTs using a pharmacological “tangle buster” of sorts might provide relief from the deleterious effects of large aggregates. Nevertheless, if fibrillar or aggregate forms of tau represent initial compensatory mechanisms to sequester more upstream oligomeric forms, then it may be that removal of this “toxic sink” would outweigh relief from tangle-associated toxicity and would be consequently more harmful than beneficial. Alternatively, small molecule inhibitors of oligomer formation or fibrillization may prevent accumulation of all toxic species at their origin. LMTX, a modified derivative of the aggregation inhibitor methylthionine chloride (methylene blue), is entering Phase III trials for bvFTD (clinical trial #NCT01626378, and see Wischik et al., 2014). Other therapeutic avenues targeting toxic species may be even more specific; immunotherapy is a potential method for the removal of pathological tau, and in several instances has been demonstrated to suppress tau pathology and improve phenotype in transgenic models of tauopathy (Asuni et al., 2007; Sigurdsson, 2008; Yanamandra et al., 2013; Castillo-Carranza et al., 2014). This strategy relies on identifying precisely the species, or epitope of the species, responsible for toxicity and successful application in human disease will rely on the resolution of this issue. If multiple toxic species exist, which is quite possible given the wide range of modifications that tau undergoes, then multiple epitopes may need to be targeted.

The primary therapeutic alternative is based on the loss of function model, making inhibition of phosphorylation and stabilization of microtubules potential therapeutic options. Numerous studies have demonstrated reduced hyperphosphorylation and aggregation of tau with alleviation of motor phenotype or axonal degeneration in murine models of tauopathy by inhibition of tau kinases such as GSK3β, PKA and PKC (Nakashima et al., 2005; Noble et al., 2005; Le Corre et al., 2006). Temsirolimus, one of the newest potential therapies for tauopathy, appears to work on two levels, through reducing tau hyperphosphorylation and increasing autophagic clearance (Jiang et al., 2014). Although there is concern regarding chronic inhibition of major regulatory kinases with an approach such as this (McKnight et al., 2012), initial studies suggest that GSK3 inhibitors such as lithium and tideglusib are well tolerated in the short term (6–12 weeks) and perhaps up to a year in patients with AD (MacDonald et al., 2008; Hampel et al., 2009; Forlenza et al., 2011; del Ser et al., 2013). Notably, lithium is already FDA-approved and could therefore avoid many regulatory steps that typically retard drug availability. Although these studies have demonstrated a lack of efficacy in AD, duration of treatment has typically been short or using low participant numbers. Microtubule stabilizing agents are currently being actively pursued, and some appear to be brain penetrant and improve microtubule density, axonal stability and cognitive performance in mouse models of tauopathy (Brunden et al., 2010a,b). However, the microtubule binding agent davunetide (NCT01110720), which promotes assembly and affords neuroprotection in a number of animal models, has recently failed in Phase III trials for PSP (Shiryaev et al., 2009; Boxer et al., 2014).

These strategies represent early therapeutic endeavors in FTLD-tau. It is common in the literature to conclude research articles by advancing new forms of tau pathology or the pathways affecting or affected by tau as potential therapeutic targets. However, it is important to note the lack of evidence for a single, dominant toxic mechanism. The complexity of FTLD-tau may be similar to that recently posed for AD, in which a heterogeneous Aβ “soup” confers toxicity, rather than a single pathogenic species (Benilova et al., 2012). In future, it will be important to refine theories and identify mechanisms that contribute most significantly to neuronal demise and that consequently have a higher chance of success in clinical trials.

## TDP-43 and TDP-43 mediated mechanisms of toxicity

### TDP-43 biology and function

Previously a relatively little-studied protein, the stature of TDP-43 has grown considerably due to description of mutations in the *TARDBP* gene in sporadic and familial cases of ALS and the discovery that TDP-43 is a major constituent of ubiquitin-positive inclusions in FTD and ALS (Neumann et al., 2006; Sreedharan et al., 2008). Subsequent demonstration that TDP-43 positive aggregates are found in numerous other neurodegenerative disorders including Parkinson’s disease and AD have consequently raised the possibility that TDP-43 may be an intriguing therapeutic target outside of FTLD-TDP (Amador-Ortiz et al., 2007; Nakashima-Yasuda et al., 2007). TDP-43 is also one of a number of disease-related proteins with a known role in RNA metabolism (Polymenidou et al., 2011), a field that over the last decade has grown considerably in impact due to an increasing appreciation for its complexity and influence in multiple biological systems.

The *TARDBP* gene encoding TDP-43 gives rise to a 414 amino acid nucleic acid binding protein containing a number of functional domains including two RNA Recognition Motifs (RRM), a glycine rich C-terminus required for binding to a host of cofactors such as members of the hnRNP family and nuclear localization and export sequences that contribute towards its ability to shuttle continuously between nucleus and cytoplasm (Buratti and Baralle, 2001; Ayala et al., 2008b; Winton et al., 2008; Ling et al., 2010). A highly mobile protein, it is redistributed from a predominantly nuclear localization to the cytoplasm in response to diverse stress stimuli (Ayala et al., 2011) such as oxidative stress (Liu-Yesucevitz et al., 2010), proteasome inhibition (van Eersel et al., 2011), endoplasmic reticulum stress (Leggett et al., 2012) and physical injury (Moisse et al., 2009) and relocalizes at synapses upon depolarization (Wang et al., 2008). Studies in humans and mice have demonstrated that TDP-43 binds a host of RNA substrates, equal to approximately 30% of transcripts in the mouse genome (Polymenidou et al., 2011). In addition to mRNA, substrates also include non-coding elements such as long noncoding, small nucleolar, small nuclear, ribosomal, micro and telomeric RNA, and in some cases binding was increased in cases of FTLD-TDP (Tollervey et al., 2011). In both mouse and human studies, reduction in TDP-43 resulted in alterations in splicing of bound transcripts (Polymenidou et al., 2011; Tollervey et al., 2011). Binding of TDP-43 to pre-mRNA with large introns results in stabilization in mice, and many of these transcripts are involved in synaptic activity (Polymenidou et al., 2011). Given that TDP-43 associates with a range of other proteins that influence microRNA processing, splicing, polyadenylation and RNA editing (Ling et al., 2010), it is likely that it is involved in a plethora of cellular functions by sheer virtue of regulating expression of transcripts in a number of ways that feed in to numerous biological pathways. This is supported by functional studies demonstrating that TDP-43 influences processes as diverse as neurite outgrowth (Iguchi et al., 2009), cell cycle dynamics (Ayala et al., 2008a), mitochondrial function (Duan et al., 2010), metabolism (Chiang et al., 2010) and autophagy (Bose et al., 2011). Evidently, TDP-43 is a critical regulator of cellular dynamics.

### TDP-43 aggregation and toxicity

Given the relatively recent discovery of TDP-43, understanding the nature of its contribution to neurodegeneration is still in its infancy. Current hypotheses regarding TDP-43 pathogenesis stem from observation of post-mortem changes in protein immunohistochemistry and biochemistry, and thus center on direct toxicity from cytoplasmic aggregates or C-terminal fragments and loss of nuclear function. These are not necessarily mutually exclusive and may act in concert. C-terminal fragments of TDP-43 are aggregate-prone, undergo phosphorylation and ubiquitination and are cytotoxic in cell culture (Igaz et al., 2009; Nonaka et al., 2009; Zhang et al., 2009). Disease linked mutations found in ALS appear to increase the production of C-terminal fragments, and their aggregation and toxicity (Johnson et al., 2009; Nonaka et al., 2009; Barmada et al., 2010). The nature of these fragments and their generation is contentious. TDP-43 can be cleaved by caspases to generate ~25 kDa and ~35 kDa fragments, and immunopositive inclusions for the caspase-cleaved conformer are found in human cases of FTLD-TDP (Zhang et al., 2007; Dormann et al., 2009), with one group suggesting that this cleavage may be neuroprotective in some way, perhaps by limiting TDP-43 activity (Suzuki et al., 2011). However, sequencing of fragments from FTLD-TDP cases has yet to identify a single consensus N-terminus amino acid for these fragments (Nonaka et al., 2009). This may be consistent with observations using phospho-specific antibodies which demonstrate multiple fragments from 17–25 kDa, the intensity of which also may be distinct for each TDP-43 subtype (Hasegawa et al., 2008). Such heterogeneity may ultimately be important given that cleavage site determines extent of phosphorylation, solubility and aggregation *in vitro* (Furukawa et al., 2011a). Although cleavage and aggregation of TDP-43 is a pathological event, such properties may also be involved in normal cellular function. Two groups have reported on the prion-like nature of the C-terminus, which self-interacts to form insoluble yet functional nuclear aggregates that are repeatedly formed and destroyed (Wang et al., 2012; Nonaka et al., 2013). Inhibiting or stabilizing this interaction is proposed to cause degradation to 25 kDa or oligomerization respectively. This segment of protein also confers structural beta-sheet conformations that appear to promote the formation of amyloid fibrils, and this propensity is enhanced by the A315T mutation found in familial ALS (Guo et al., 2011). Thus, function and pathogenicity may be two sides of the same coin, and the prion nature of TDP-43 opens up the intriguing possibility of prion-like spreading of disease (Nonaka et al., 2013). One group has further suggested that prion-like aggregation of TDP-43 or C-terminal fragments may be modulated by a dynamic interaction with heat shock proteins (Udan-Johns et al., 2014).

### TDP-43 loss of function and toxicity

At least three studies using aggregate-prone C-terminal fragments or *in vitro-*generated fibrillar TDP-43 have demonstrated that aggregates are capable of sequestering the full length protein, and therefore depleting endogenous nuclear function (Nonaka et al., 2009; Che et al., 2011; Furukawa et al., 2011b), although this is not true for all studies (Zhang et al., 2009). Consequently, the reason for nuclear depletion is not fully understood, but is likely to be deleterious given that numerous studies *in vitro* and *in vivo* suggest TDP-43 levels necessitate tight regulation. Overexpression and knockdown *in vitro* can confer toxicity (Ayala et al., 2008a; Suzuki et al., 2011) and most persuasively knockout *in vivo* results in lethality. This occurs both in conditional knockout animals in which deletion is postponed until adulthood and *in utero* in constitutive *Tardbp*^–/–^ mice (Chiang et al., 2010; Kraemer et al., 2010; Sephton et al., 2010; Wu et al., 2010). Loss of TDP-43 specifically in motor neurons results in cell death and an ALS-like phenotype in mice (Wu et al., 2012) and reduced TDP-43 expression in *Drosophila* and zebrafish results in motor deficits (Feiguin et al., 2009; Kabashi et al., 2010). These data suggest that TDP-43 may be a critical regulator of cellular function and pathological loss of function may have severe consequences for cellular integrity. Furthermore, loss of TDP-43 activity may result in altered response to cellular stress, as reduced TDP-43 levels results in reduction in the number and size of stress granules (SGs), transient cytoplasmic bodies involved in RNA triage that form in response to oxidative stressors (McDonald et al., 2011). Reduction of TDP-43 in primary neuronal cell culture also increases sensitivity to toxic proteasome inhibition (van Eersel et al., 2011). Both of these cellular insults may be involved in the initial formation of TDP-43 pathology. SGs, which contain TDP-43 itself, may form the initial “seeds” for subsequently greater aggregation whilst proteasomal inhibition also induces nuclear clearance and cytoplasmic aggregation of TDP-43 *in vivo* and *in vitro* (Liu-Yesucevitz et al., 2010; Meyerowitz et al., 2011; Parker et al., 2012; Tashiro et al., 2012).

Thus, as for tau, there are potentially a number of different mechanisms by which TDP-43 dysfunction contributes to neuronal demise, and which may not be mutually exclusive. Recently the tau field has reached a point of equilibrium with regards to the loss of function vs. gain of toxicity debate. It will be intriguing to see in the near future if there is a definitive answer for TDP-43 proteinopathies.

## Therapeutic strategies for FTLD-TDP

Understanding of TDP-43 proteinopathy or even TDP-43 endogenous function is currently limited and thus so are therapeutic expectations. However, plausible logical suggestions would be restoration of nuclear function and reduction in formation of or increased clearance of aggregates. As loss of TDP-43 has significant implications for cellular integrity, aiming to reduce expression and therefore aggregate formation, as proposed for tau, is a conceptual non-starter. One may hypothesize that preventing cytoplasmic accumulation and retaining nuclear function may prove beneficial. In this case, simply defining the mechanism by which loss of nuclear function occurs is critical. If it is underpinned by cytoplasmic aggregate sequestration, then targeting aggregated material would logically provide relief from both nuclear loss and any direct cytotoxicity. At least *in vitro* dimebon and methylene blue, two compounds that were successful in Phase II clinical trials for AD, are capable of reducing TDP-43 aggregation (Yamashita et al., 2009). Currently, our knowledge of TDP-43 biology is probably too limited to make TDP-targeted therapies a realistic short term goal. Increased knowledge of basic function and the development of more sophisticated study tools such as animal models recapitulating TDP-43 pathology and patient specific induced pluripotent stem cells (iPSCs) should provide a greater understanding of pathways amenable to therapy (Bilican et al., 2012; Egawa et al., 2012). Indeed, iPSCs have already been used to screen drug candidates, identifying anacardic acid as a potential modifier of insoluble TDP-43 species (Egawa et al., 2012).

## Progranulin as an unbiased therapeutic candidate

For both tau and TDP-43 directed therapies, there is a broader question raised by the presence of multiple disease subtypes (for example, CBD, PSP and PiD in FTLD-tau and type A-D for FTLD-TDP). As the pathology for each disease differs, the mechanisms leading to their formation may also be dissimilar. Would a different therapeutic strategy be required for each, or is there a keystone mechanism underpinning them all? This question is analogous to that posed in the cancer field where optimal breast cancer treatment paradigms depend on the type of cancer present (Kelly and Buzdar, 2013). A therapy that could be used more globally and that is not dependent on the type of pathology might therefore have greater application. The discovery of loss-of-function mutations in progranulin as a cause of TDP-43 proteinopathy may provide such a target. When compared with the challenge of identifying toxicity due to tau and TDP-43, raising levels of the potentially neuroprotective progranulin is a conceptually simple solution. Recent identification of TNF (Tang et al., 2011) [although this interaction is disputed (Chen et al., 2013)] and sortilin (Zheng et al., 2011) receptors that bind progranulin offer potentially modifiable and druggable targets. Indeed, small molecule inhibition of sortilin-mediated endocytosis restores extracellular progranulin levels in iPSCs derived from progranulin mutation carriers to wild type levels (Lee et al., 2013a). Additionally, chemicals that upregulate progranulin levels through enhanced transcription (suberoylanilide hydroxamic acid) or the stabilization of intra- and consequently extracellular progranulin by the alkalization of intracellular compartments have been identified (chloroquine, bepridil, and amiodarone), some of which are already FDA-approved (Capell et al., 2011; Cenik et al., 2011). Finally, protective genetic variants of the transmembrane protein *TMEM106b* were identified in a genome wide association study of *GRN* mutation carriers. These variants delay age of onset or reduce penetrance in *GRN* mutation carriers and correlate with plasma progranulin levels, implicating *TMEM106b* or interacting pathways as therapeutic targets (Van Deerlin et al., 2010; Cruchaga et al., 2011; Finch et al., 2011). The applicability of these or other future progranulin therapies to neurodegenerative diseases outside of FTLD-TDP could be tested *in vivo* in animal models, with transgenic models of tauopathy/FTLD-tau an obvious candidate.

A number of challenges present themselves. Primarily, little is known regarding how progranulin is processed in the brain, with the majority of knowledge coming from studies in the periphery. In this environment, the progranulin precursor—composed of seven and a half repeats of a common 12-cysteine module called granulin—can be processed proteolytically by elastase and proteinase 3 to produce the individual granulins (Kessenbrock et al., 2008), which may have distinct effects on biological processes. At present, it is unknown whether the same mechanisms occur in the brain or whether loss of full-length progranulin or individual granulin(s) is the cause of FTLD in familial forms. At least two studies have suggested that the neurotrophic properties of progranulin may be due to module granulin activity rather than the full-length form (Gass et al., 2012; De Muynck et al., 2013). If distinct biological effects exist for the full-length progranulin precursor and each individual granulin, the results of elevating the former in FTD patients may be a complex and multifaceted mix of therapeutic and unintended side effects. It is consequently imperative to either deconstruct the desirable mechanisms underlying progranulin function or to demonstrate *in vivo* the safety, tolerability and physiological benefits of increased progranulin levels. Encouragingly, mice overexpressing progranulin are significantly rescued from the behavioral deficits induced by middle cerebral artery occlusion stroke paradigms coincident with a shift to a more anti-inflammatory profile in transgenic animals (Tao et al., 2012; Egashira et al., 2013). This shift in inflammatory profile is consistent with a role for progranulin in modulating neuroinflammatory responses (Cenik et al., 2012; Martens et al., 2012; Tanaka et al., 2013). Progranulin knockout mice—a model of inherited FTLD due to *GRN* mutation—provide a useful tool for studying progranulin deficiency but lack overt TDP-43 aggregation and nuclear clearance as observed in humans (Ahmed et al., 2010). Pathology is restricted to hyperphosphorylated, insoluble TDP-43 that remains largely nuclear (Wils et al., 2012). Such tools may be necessary to fine tune elevation of progranulin levels, given its potential role in tumorigenesis (Tangkeangsirisin and Serrero, 2004; Matsumura et al., 2006; Göbel et al., 2013). In sum, progranulin therapy is an attractive target for FTLD-TDP, particularly so for families with heritable progranulin mutations, but gaps remain in our understanding of disease that may need to be resolved.

## Diagnostic progress and the hurdle of heterogeneity

### Current clinical, imaging and biochemical diagnostics

Of equal importance in therapeutic consideration is the ability to diagnose individuals with specific disease. In this regard, neuropathology remains the dominant driver of disease mechanism theory and consequently therapeutic hypotheses, but determination of underlying FTLD-tau or FTLD-TDP early in the disease process will be essential in any therapeutic endeavor. As alluded to above, hypothetical therapies may also need to be designed to individual subtypes of TDP and tau pathology, if no singular process can be commonly delineated or effectively targeted. There are a number of diagnostic approaches under consideration that can be categorized as clinical, biological and those using imaging technologies.

**Figure 2:**
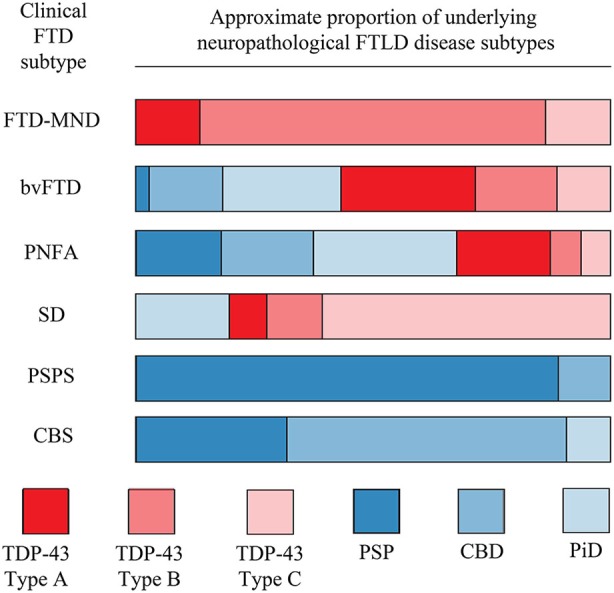
**Relationship between clinical FTD phenotype and underlying FTLD disease neuropathology**. For each clinical subtype on the left, the bar is divided to show the approximate proportion of cases which are underpinned by the indicated disease neuropathology. Profound neuropathological heterogeneity exists for many clinical subtypes, particularly bvFTD and PNFA. This figure is based on data from Josephs et al. (2011).

Clinical-pathological studies of FTD have advanced rapidly, resulting in several changes in neuropathologic diagnostic and nosologic criteria for FTLD to reflect these findings (Cairns et al., 2007). There are probabilistic correlations between the syndromes and diseases underlying them, which invites some optimism in disease and subtype diagnosis in symptomatic individuals (Figure 2; Josephs et al., 2011). SD and FTD-MND are almost exclusively FTLD-TDP whereas PSPS and CBS are almost always characterized by tau pathology. PNFA and bvFTD are more neuropathologically heterogeneous, which is unfortunate given the high prevalence of bvFTD (in the largest clinicopathological study to date, bvFTD represented around 45% of FTD). Furthermore, in some cases there are correlations—albeit weaker ones—between specific subtypes of tau or TDP-43 pathology and clinical phenotype. For example, FTD-MND is not only purely FTLD-TDP but is approximately 70% type B and TDP-43 positive SD almost 85% type C (Josephs et al., 2011). Again, bvFTD is a much more complex syndrome in terms of either TDP-43 or tau subtype. However, one intriguing suggestion is that FTLD-TDP and FTLD-tau may affect distinct, distant brain networks and that detailed neuropsychological analysis may be used to differentiate the network—regardless of the syndrome—and hence pathology (Grossman et al., 2008; Listerud et al., 2009; Seeley et al., 2009).

While such correlations are interesting on a population level, there is clear heterogeneity in disease/syndrome pairing and often overlap between syndromes, meaning there is currently minimal utility in diagnosing pathology in the clinic for individual patients based solely on phenotype. Furthermore, potentially the greatest issue for FTLD therapies—and neurodegenerative disease in general—is that of timing of intervention. Clinical trials in AD have generally reached the conclusion that the current pharmacological therapies in trial may require earlier administration if a clinical benefit is to be achieved. This may require studies in pre-symptomatic patients, as the appearance of pathology predates the manifestation of clinical symptoms by some time, potentially as much as 15–20 years (Golde et al., 2011; Bateman et al., 2012). Trials targeting earlier in AD now depend upon the use of diagnostic screening to identify patients at risk on the basis of preclinical disease biomarkers (Vellas et al., 2013). Although the length of presymptomatic phase may differ, this is distinctly probable in other chronic neurodegenerative diseases such as FTLD (Jacova et al., 2013), thus the early identification of people at risk of disease becomes crucial and will be underpinned by either biochemical and/or imaging techniques.

Antemortem neuroimaging analysis using the Aβ plaque binding radioligands florbetapir and Pittsburgh compound B have provided tremendous insight into pre-symptomatic disease progression in AD (Klunk et al., 2004; Bateman et al., 2012; Clark et al., 2012). Although the field currently lacks a substrate specific ligand for TDP-43, the recent development of a class of tau ligands will surely expedite studies in prodromal tauopathy patients (Maruyama et al., 2013). In the meantime, other imaging markers such as atrophy of specific brain regions are proving on the group level to correlate well with syndrome (Hu et al., 2011) and have been used, for example, to demonstrate that there is phenotypic heterogeneity linked to distinct atrophic patterns even within a subgroup such as bvFTD (Whitwell et al., 2009b). Specifically relating to disease prediction, results are currently inconclusive as to whether atrophy can predict FTLD-tau or FTLD-TDP pathotype (Grossman et al., 2007; Kim et al., 2007; Pereira et al., 2009; Whitwell et al., 2009a), although it appears that TDP-43 subtypes may be associated with distinct regional atrophy once FTLD-TDP is neuropathologically confirmed (Whitwell et al., 2010). These and other sophisticated techniques such as arterial spin labeling (Hu et al., 2010b) and 18F fluorodeoxyglucose positron emission tomography (Womack et al., 2011) may be useful in the future for diagnosing underlying disease or for following disease progression in therapeutic trials. However, as a diagnostic tool in screening a presymptomatic population, they are highly impractical due to expense and time consumption.

Ideally, the identification of biochemical biomarkers for the discrimination of FTLD-tau or FTLD-TDP would provide a more cost effective and less time consuming alternative to imaging and work has begun in probable cohorts based on syndromic presentation and pathologically confirmed cases of FTLD. Although TDP-43 appears to be raised in either plasma or CSF of clinically defined FTD or FTD-MND populations, there is significant overlap with control or AD subjects and it remains unclear if such an approach will prove clinically useful (Foulds et al., 2008; Steinacker et al., 2008). Similarly, results to date suggest that total tau or tau phosphorylated at threonine 181 may not be good candidates for discriminating FTLD-tau from control (Vanmechelen et al., 2000; Bian et al., 2008; Kapaki et al., 2008). Using unbiased proteomic approaches, it appears that FTLD-TDP and FTLD-tau might be distinguished from one another on the basis of several CSF analytes, although the specificity and reliability of these remain to be determined in larger studies (Hu et al., 2010a). It is also unknown whether the heterogeneity of TDP-43 pathology (subtypes A-C) and FTLD-tau (PiD, CBS, PSP) can be disaggregated at the biochemical level. For example, it appears PSP may have a unique truncated tau profile in CSF that separates it from other tau diseases (Borroni et al., 2009b) although this has not been replicated in additional, independent studies (Kuiperij and Verbeek, 2012).

### Towards multidimensional, presymptomatic diagnostics

While these studies represent early forays into biomarker evaluation, more powerful proteomic screening of CSF or plasma from clinically predicted FTLD-tau and FTLD-TDP may ultimately provide better biomarker possibilities and will require follow-up in larger, longitudinal cohorts to test their sensitivity and specificity. As such development occurs, it should do so with the heterogeneity of FTLD in mind and potentially involve other neurodegenerative diseases. Although one type of neuropathology may be dominant and one brain region afflicted more than others, cases of autopsy-confirmed FTLD may be further complicated by concomitant Aβ, vascular or alpha synuclein pathology (National Institute on Aging, 1997; McKeith et al., 2005; Toledo et al., 2013). This is already apparent in some biomarker studies in which perhaps as many as 30% of clinical FTLD patients are also AD biomarker positive (Schoonenboom et al., 2012) and in diagnostic imaging assays used for dementia with Lewy bodies (DLB) where there may be significant overlap with FTD (Morgan et al., 2012). In addition to AD and DLB, TDP-43 or phospho-TDP-43 pathology can be found co-occurring with tau pathology in cases of CBD, PSP, PiD and argyrophilic grain disease (Higashi et al., 2007; Uryu et al., 2008; Arai et al., 2009; Fujishiro et al., 2009; Rohn and Kokoulina, 2009; Yokota et al., 2010).

**Figure 3:**
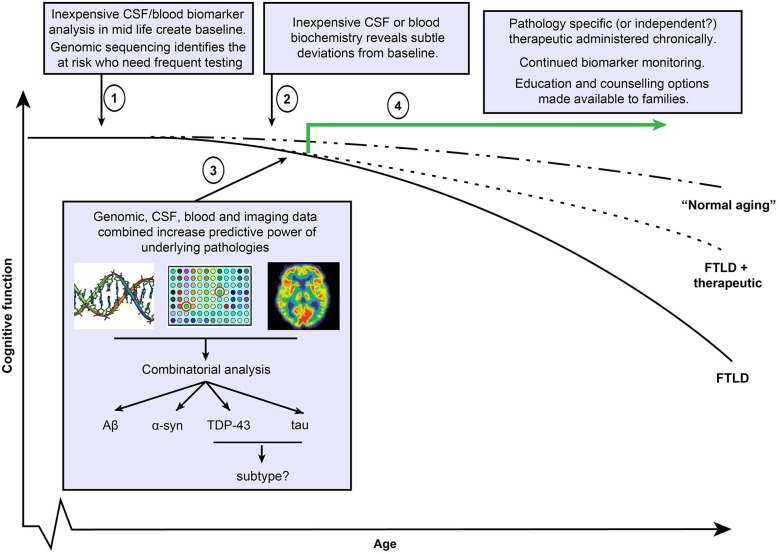
**Potential FTLD diagnostic and therapeutic timeline**. Clinical trials in Alzheimer’s disease (AD) suggest that therapeutics will be maximally effective when administered in early disease course. (1) Under this scenario, simple and economically viable blood or cerebrospinal fluid based screens at regular age intervals establish an individual’s biomarker status at cognitively healthy baseline and also with respect to general population. Screens include markers of all known disease pathologies including Aβ, α-syn, TDP-43 and tau. Inexpensive genomic sequencing may help to identify at risk individuals who require more regular evaluation. (2) As pathology (or multiple pathologies) progress, regular screening identifies deviations from the baseline that could potentially indicate early stage disease, triggering more frequent evaluations. (3) Continued biomarker positivity or progression justifies the use of more expensive imaging methods to determine regional atrophy or using pathology binding agents (such as florbetapir or as yet incompletely characterized tau binding agents). Such data, when combined with CSF, blood and potentially genetic alleles that associate with pathologies or sub types of pathology, may provide potentially more predictive power than a reliance on a single method. (4) Administration of single or multiple therapeutics depending on results, continued follow up evaluations to test response to treatment. As some individuals may be unresponsive to treatments, and future therapeutics may not entirely halt disease progression, social support structures including education and counselling for individuals and families should be integrated into care.

As a whole, these findings suggest that (1) clinical symptoms are not wholly reliable as an indicator of an underlying pathology or disease to be targeted for treatment; (2) an underlying disease pathology at autopsy is not a guaranteed predictor of a clinical phenotype; and (3) mixed pathologies and therefore diseases are often coexistent. Additionally, moving earlier in disease course towards “cognitively normal”—as will be the desired course for earlier administration of therapies—will increasingly blur the diagnostic line between “normal” and disease, creating greater chance of false positives or negatives. This “messy reality” of neurodegenerative disease means that a search for a single, definitive disease diagnosis in individuals will probably never achieve the same level of certitude as for diseases such as viral or bacterial agents. That being said, with the advancing sophistication of technology and data collection in medicine and the decreasing cost of genome sequencing, it is not unrealistic to envisage a combination of all or some of blood, CSF, and genetic “panels” that contain large numbers of analytes or alleles known to associate with different neurodegenerative pathology. Initial probable diagnoses could be refined by subsequent imaging studies. Such a multifaceted approach to disease diagnosis would certainly have far greater power than those relying on singular readouts, and may be the only way to discriminate early stage disease from “normal aging” as small deviations from the norm across multiple analytes synergize to create disease pattern readout (Figure 3). However, such an approach would still only return probabilistic diagnoses and has many considerations. Identifying individuals at risk who should undergo diagnostic examination is not a trivial issue. Determining who is screened, when, and how often, both in trials and using any eventual treatment identified is a priority. This may be based on a mixture of risk factors, including age, family history, common place biochemical screens, genetic risk alleles, or environmental risk factors that are currently unstudied (Onyike and Diehl-Schmid, 2013). Alternatively, suspending testing until clinical symptoms manifest may be too late in disease progression. Currently, we do not know at what stage intervention might be required and determining this by trial and error will be time consuming and financially costly. Furthermore, decades of unsuccessful clinical trials would also exact a human toll, and for this reason, it is important that social support resources be available to patients and families. Undoubtedly, clinical trials will necessitate great foresight and planning and such considerations will need to be debated prior to implementation of therapeutics.

## Conclusion

As outlined above, the ability to use biomarkers to identify specific subtypes of underlying FTLD-TDP or FTLD-tau in early stage disease (whether prodromal or asymptomatic phases) is a challenging issue, but one of paramount importance if the experiences of AD clinical trials in symptomatic patients also extrapolate to FTLD. Further complication arises regarding the complexity of disease. At the outset of studies focusing on tau, the prevailing theory was that removal of NFTs would provide a therapeutic breakthrough. Years on, that notion seems oversimplified and tauopathies are recognized as extremely complex, with an abundance of biochemical and structural tau alterations that may all, in some form or other, contribute to neuronal demise through mechanisms that are as yet not fully understood. Furthermore, such processes may not be a single linear mechanism, both in the formation of detrimental tau species and in their effect. It is inevitable that increased study will reveal an even greater complexity and this is highly probable for FTLD-TDP also. Although this means that we are increasingly knowledgeable about neurodegenerative mechanisms, it also suggests that these are multifactorial, multi-mechanistic diseases that current technology may find difficult to address. In this respect, it is possible that in addition to the “knowledge gaps” highlighted above, we may face somewhat of a “technology gap”. Compared to the myriad biochemical changes in tau, TDP-43 and other cellular processes, all of which occur in different cell types at various stages between healthy and degenerating, delivery of a single pharmaceutical compound aimed at a sole process or pathway to the whole brain may be oversimplified. The invention of novel technologies or use of multiple, simultaneous therapeutic strategies may be required. In the interim, care must be taken in the selection of therapeutics going forward as financing of trials for therapeutic interventions will not continue *in aeternum* if strategies based on unproven mechanisms fail repeatedly in clinical trials.

## Conflict of interest statement

The authors declare that the research was conducted in the absence of any commercial or financial relationships that could be construed as a potential conflict of interest.
